# Photon Hunting in the Twilight Zone: Visual Features of Mesopelagic Bioluminescent Sharks

**DOI:** 10.1371/journal.pone.0104213

**Published:** 2014-08-06

**Authors:** Julien M. Claes, Julian C. Partridge, Nathan S. Hart, Eduardo Garza-Gisholt, Hsuan-Ching Ho, Jérôme Mallefet, Shaun P. Collin

**Affiliations:** 1 Laboratoire de Biologie Marine, Earth and Life Institute, Université catholique de Louvain, Louvain-la-Neuve, Belgium; 2 School of Biological Sciences, University of Bristol, Bristol, United Kingdom; 3 Neuroecology Group, School of Animal Biology and the UWA Oceans Institute, The University of Western Australia, Crawley, Australia; 4 National Museum of Marine Biology and Aquarium, Checheng, Taiwan; 5 Institute of Marine Biodiversity and Evolutionary Biology, National Dong Hwa University, Shoufeng, Taiwan; University Zürich, Switzerland

## Abstract

The mesopelagic zone is a visual scene continuum in which organisms have developed various strategies to optimize photon capture. Here, we used light microscopy, stereology-assisted retinal topographic mapping, spectrophotometry and microspectrophotometry to investigate the visual ecology of deep-sea bioluminescent sharks [four etmopterid species (*Etmopterus lucifer*, *E. splendidus*, *E. spinax* and *Trigonognathus kabeyai*) and one dalatiid species (*Squaliolus aliae*)]. We highlighted a novel structure, a translucent area present in the upper eye orbit of Etmopteridae, which might be part of a reference system for counterillumination adjustment or acts as a spectral filter for camouflage breaking, as well as several ocular specialisations such as aphakic gaps and semicircular tapeta previously unknown in elasmobranchs. All species showed pure rod hexagonal mosaics with a high topographic diversity. Retinal specialisations, formed by shallow cell density gradients, may aid in prey detection and reflect lifestyle differences; pelagic species display areae centrales while benthopelagic and benthic species display wide and narrow horizontal streaks, respectively. One species (*E. lucifer*) displays two areae within its horizontal streak that likely allows detection of conspecifics' elongated bioluminescent flank markings. Ganglion cell topography reveals less variation with all species showing a temporal area for acute frontal binocular vision. This area is dorsally extended in *T. kabeyai*, allowing this species to adjust the strike of its peculiar jaws in the ventro-frontal visual field. *Etmopterus lucifer* showed an additional nasal area matching a high rod density area. Peak spectral sensitivities of the rod visual pigments (λ_max_) fall within the range 484–491 nm, allowing these sharks to detect a high proportion of photons present in their habitat. Comparisons with previously published data reveal ocular differences between bioluminescent and non-bioluminescent deep-sea sharks. In particular, bioluminescent sharks possess higher rod densities, which might provide them with improved temporal resolution particularly useful for bioluminescent communication during social interactions.

## Introduction

Located between the bright epipelagic and dark bathypelagic zones, the mesopelagic twilight zone (200–1000 m) consists of a visual scene continuum where, with increasing depth, extended down-welling sunlight is progressively replaced by point-like bioluminescent emissions [1]. In this vast dim habitat, however, many animals rely on vision for their survival and hence have evolved various strategies to optimize photon capture [2]. Mesopelagic fishes in particular, have developed an impressive array of ocular adaptations, including large and/or upward/forward-pointing tubular eyes [2], [3], aphakic gaps [4], [5], wide immobile pupils [6], long photoreceptor outer segments and/or multibank retinae [7], [8], single-visual pigment rod photoreceptors (usually shortwave-sensitive) [9], reflective tapeta [10], and high convergence ratios between photoreceptors and ganglion cells (i.e. high spatial summation) [11], in order to increase optical sensitivity. Higher sensitivity can only be achieved at the detriment of acuity (spatial resolution) [12]. Nevertheless, some large mesopelagic fish species have almost totally escaped this constraint by having gigantic eyes with long focal lengths, which allow both high sensitivity and sharp resolution [11]. In addition, many other fishes have partially resolved this trade-off by displaying heterogeneous retinae, with some parts devoted to high sensitivity and other parts mediating high resolution. Far from requiring complex accessory structures, this heterogeneity is only achieved by a variation in the spatial summation of photoreceptors onto ganglion cells [2]. In extreme cases, the retina shows a deep convexiclivate fovea, where ganglion cells are densely packed into a pit in order to allow precise localization of point sources of bioluminescence [1], [8], [13].

Retinal ganglion cell topography has been found to reflect the habitat and ecology of deep-sea species, which are always challenging to study whether in the wild or in captivity [11], [14], [15]. Ganglion cells either form areae, such as an ‘*area centralis*’ (centripetal density gradient) which (often) facilitates binocular vision and the targeting of a precise region in a complex three-dimensional environment [8], [13], or a ‘visual streak’ that provides a panoramic view of a horizontal habitat, typically the sand-water (benthic species) or water-air interface (pelagic species) [13], [18], [19]. Many species display more than one specialisation [8], [13], and the retinal acute zones may take various forms including arches [20] or rings [21]. Such zones are also found at the level of the photoreceptor layer, where they usually match the topographic distribution of ganglion cells. However, photoreceptor and ganglion cell topographies may not always co-register, which (for duplex retinae) may be a consequence of a visual shift between scotopic and photopic conditions [22]. It might also reveal a trade-off between sensitivity and temporal resolution. Indeed, the temporal response properties (visual processing speed) of a photoreceptor is inversely proportional to its outer segment volume [23],[24],[25]. This implies that (in retinae with homogeneous photoreceptor outer segment lengths) a part of the visual field subtended by a high-density area of photoreceptors will theoretically be sampled with a higher temporal resolution, which allows the detection of fast moving objects [26].

Lanternsharks (Etmopteridae) and kitefin sharks (Dalatiidae) are small [usually less than 50 cm in total length (TL)] bioluminescent elasmobranch fishes that occupy numerous deep-sea biotopes, sometimes in very high numbers. Although they represent ∼12% of currently known shark species and are key predators of many oceanic communities, their biology and ecology is poorly known [27]. It is assumed that most of them perform vertical migrations and use their hormonally-controlled photogenic organs (photophores), whose intrinsic chemistry remains mysterious [28], to disguise their silhouette in the water column when viewed from below, a common pelagic camouflage tactic called counterillumination and used by many species [29], [30], [31], [32], [33]. Many lanternsharks are also thought to use their photophores for bioluminescent signalling, either to facilitate intraspecific behaviours [34], [35], [36], via clade-specific lateral markings, or to highlight the presence of their defensive finspines (an interesting example of bioluminescent aposematism) [37]. Due to obvious logistical difficulties, no behavioural data either from wild or experimental animals are currently available to support these hypotheses.

**Figure 1:**
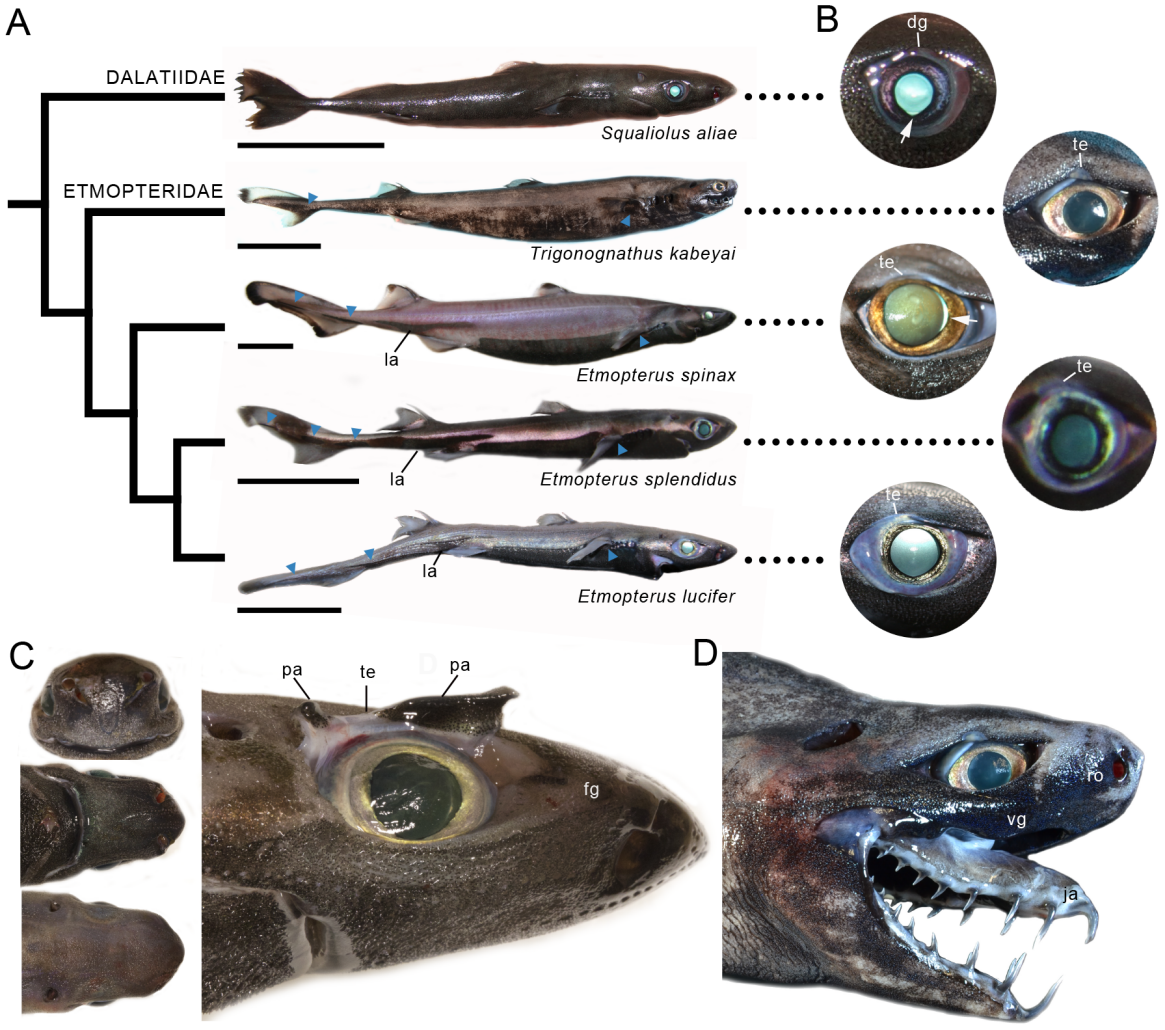
External body and ocular features. (A) Phylogeny of sharks analysed in this study (from [89]). Lateral pictures of representative specimens indicate the position of the clade-specific bioluminescent lateral markings (la) and other bioluminescent areas probably involved in intraspecific behaviours (blue triangles). Scale bars, 5 cm. (B) Close-up of the eyes showing the position of aphakic gaps (white arrow) and translucent upper eyelid (te) or dorsal groove (dg) in some species. (C) Frontal (top left), ventral (middle left), dorsal (down left) and lateral (right) views of *E. spinax* head showing the part of the visual field subtended by the eyes. Note the presence of a pronounced frontal groove (fg) favouring frontal binocular vision. The dissected upper orbital region shows the translucent eyelid area (te) is delimited caudally and frontally by aggregations of photophores (pa) pointing towards the eye. (D) Head of *T. kabeyai* with protruded jaws (ja). Note how binocular vision is prevented frontally by an enlarged rostrum (ro) and facilitated ventrally (towards the end of the jaw) by a ventral groove (vg).

Here, we investigate the visual system of five bioluminescent shark species, including the elusive viper dogfish (*Trigonognathus kabeyai*) known from <50 specimens [38], and which demonstrate a high phylogenetic, ecological and morphological diversity (Fig. 1A). We also aim to provide a unique glimpse into the visual world of one of the most enigmatic groups of mesopelagic sharks. Using morphological analyses of ocular structures combined with topographic mapping (photoreceptors and ganglion cells) and microspectrophotometry (MSP), we describe a number of unique visual adaptations reflecting the interplay between the production and perception of the bioluminescent glows that are emitted by these inhabitants of the twilight zone. We also compare the visual characteristics of members of the Etmopteridae and Dalatiidae with those of deep and shallow-living non-bioluminescent sharks to give new insights into the evolutionary drivers of shark visual performance.

## Results

### External ocular features

Bioluminescent shark species investigated in this study display lateral camera-type eyes with large immobile pupillary apertures, spherical lenses and a high diversity of tapetal reflectance, iris coloration (blue, yellow or orange with a varying degree of dark pigmentation) and relative eye size (Figure 1B). Ventral and nasal aphakic gaps are observed in *S. aliae* and *E. spinax*, respectively; the ventral aphakic gap of *S. aliae* is accompanied by a triangular ventral slit in the iris. All etmopterids (especially *T. kabeyai*) harbour a translucent area in the middle region of the upper orbit, while this area is occupied by a dorsal groove in the Dalatiidae i.e. *S. aliae* (Figure 1B). Interestingly, the area around this translucent tissue, which appear to be an extension of the skin surrounding the eyes, is edged by numerous photophores directed into the eye and hence toward the retinal photoreceptors (Figure 1C). External morphology suggests that all species have a large visual field with regions of potential dorsal, ventral and frontal binocular overlap (Figure 1C), except *T. kabeyai*, which possesses a very thick rostrum that prevents frontal vision (Figure 1D).

### Internal ocular features

**Figure 2:**
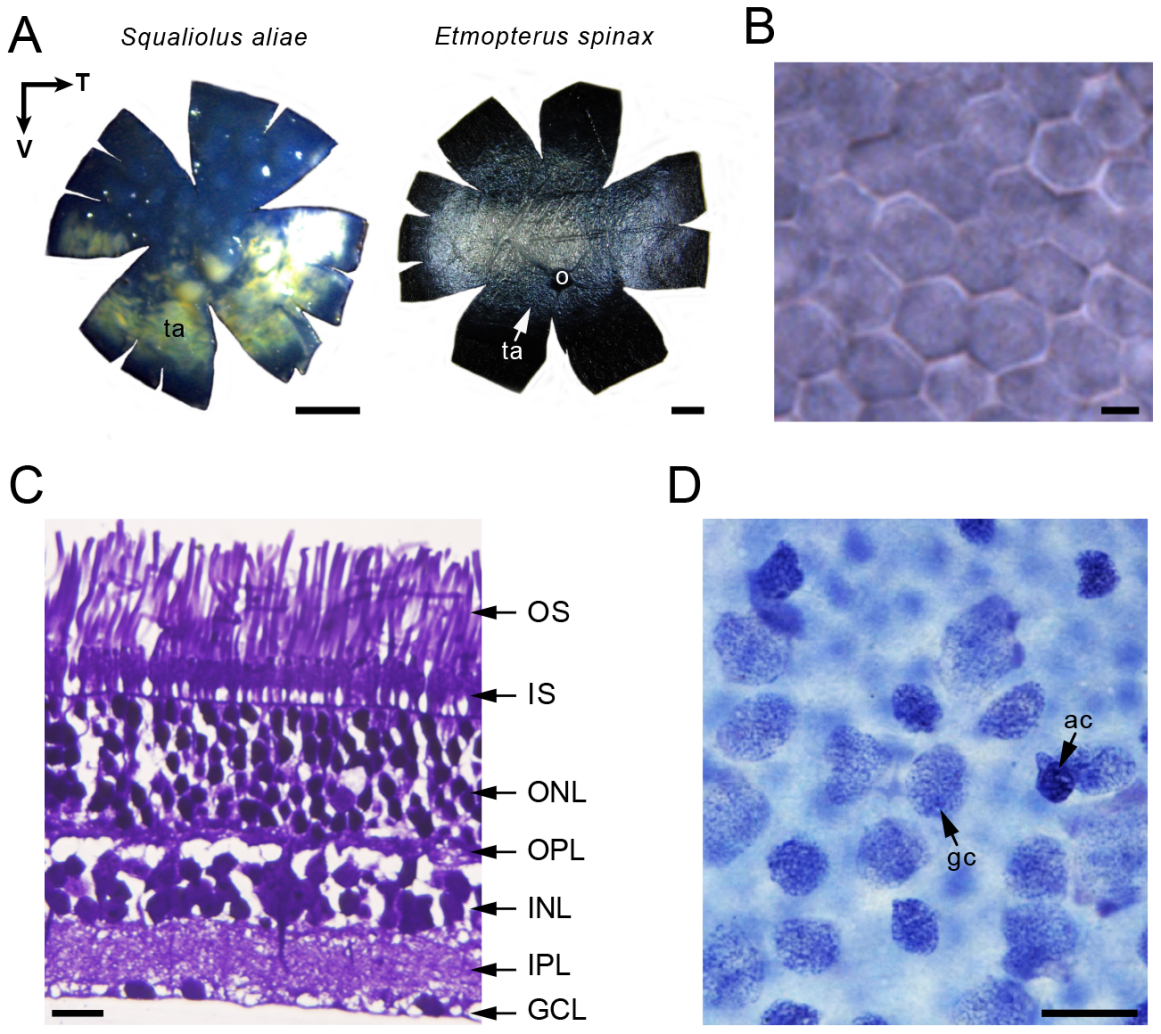
Internal ocular features. (A) Ventral (left) and horizontal (right) choroidal tapeta (ta). Photographs were taken with (ventral tapetum) or without the retina (horizontal tapetum). Black arrows indicate retina/choroid orientation (N = nasal, V = ventral). o, optic nerve. Scale bars, 2 mm. (B) Retinal hexagonal photoreceptor mosaic (wholemount view). Scale bar, 2 µm. (C) Light micrographs of transverse section through the retina of two bioluminescent shark species showing variation in photoreceptor outer segment (OS) length and diameter. GCL, ganglion cell layer; INL, inner nuclear layer; IPL, inner plexiform layer; IS, photoreceptor inner segment; ONL, outer nuclear layer; OPL, outer plexiform layer. Scale bar, 20 µm. (D) Light micrographs of the Nissl stained GCL of *E. splendidus* (wholemount view; temporal area). ac, amacrine cell; gc, ganglion cell. Scale bar, 20 µm.

The choroidal tapetum occupies a central position in etmopterid retinas, while it is present only ventrally in *S. aliae* (Figure 2A); it always showed a silver colour. All species possess pure rod photoreceptor retinas organised as hexagonal mosaics (Figure 2B). Histological sections in the central retina reveal that these rods are morphologically different across species (Table 1) – unfortunately, no retinal tissue from *T. kabeyai* was available for this analysis since the sole available retina of this species was used for retinal mapping. Rods have very long cylindrical outer segments that range from ∼50 µm in *S. aliae* to ∼70 µm in *E. splendidus*. The photoreceptor layer is single banked and comprises 30.51–38.29% of the whole retinal thickness (Table 1, Figure 2C); this retinal thickness appears quite uniform across the retina. Characterised by a sparsely populated inner retina, the ganglion cell layer of all species is largely dominated by ganglion cells with few ‘displaced’ amacrine cells observed (Figure 2D).

**Table 1:** Ocular measurements.

Species	TL (cm)	Eye Ø (mm)	Lens Ø (mm)	IPL (µm)	INL (µm)	OPL (µm)	ONL (µm)	RIS length* (µm)	ROS length* (µm)	ROS Ø* (µm)
*Etmopterus lucifer*	46.60^F^	2.98^R^	6.42	27.59	24.55	27.59	36.26	20.50	51.47	2.47
*Etmopterus spinax*	41.50^M^	16.35^R^	6.95	43.67	22.45	8.33	66.4	19.13	51.18	3.58
*Etmopterus splendidus*	21.20^F^	7.65^R^	3.63	43.95	39.35	13.9	79.06	22.13	69.35	2.16
*Squaliolus aliae*	20.50^M^	6.68^R^	3.06	39.49	40.38	10.41	62.19	18.51	48.43	2.77

*These data correspond to mean of 10 measurements from the central retina of a single specimen. F, female; INL, inner nuclear layer; IPL, inner plexiform layer; L, left eye; M, male; ONL, outer nuclear layer; OPL, outer plexiform layer; R, right eye; ROS, rod outer segment; RIS, rod inner segment; TL, total length.

### Topographic specialisations

**Figure 3:**
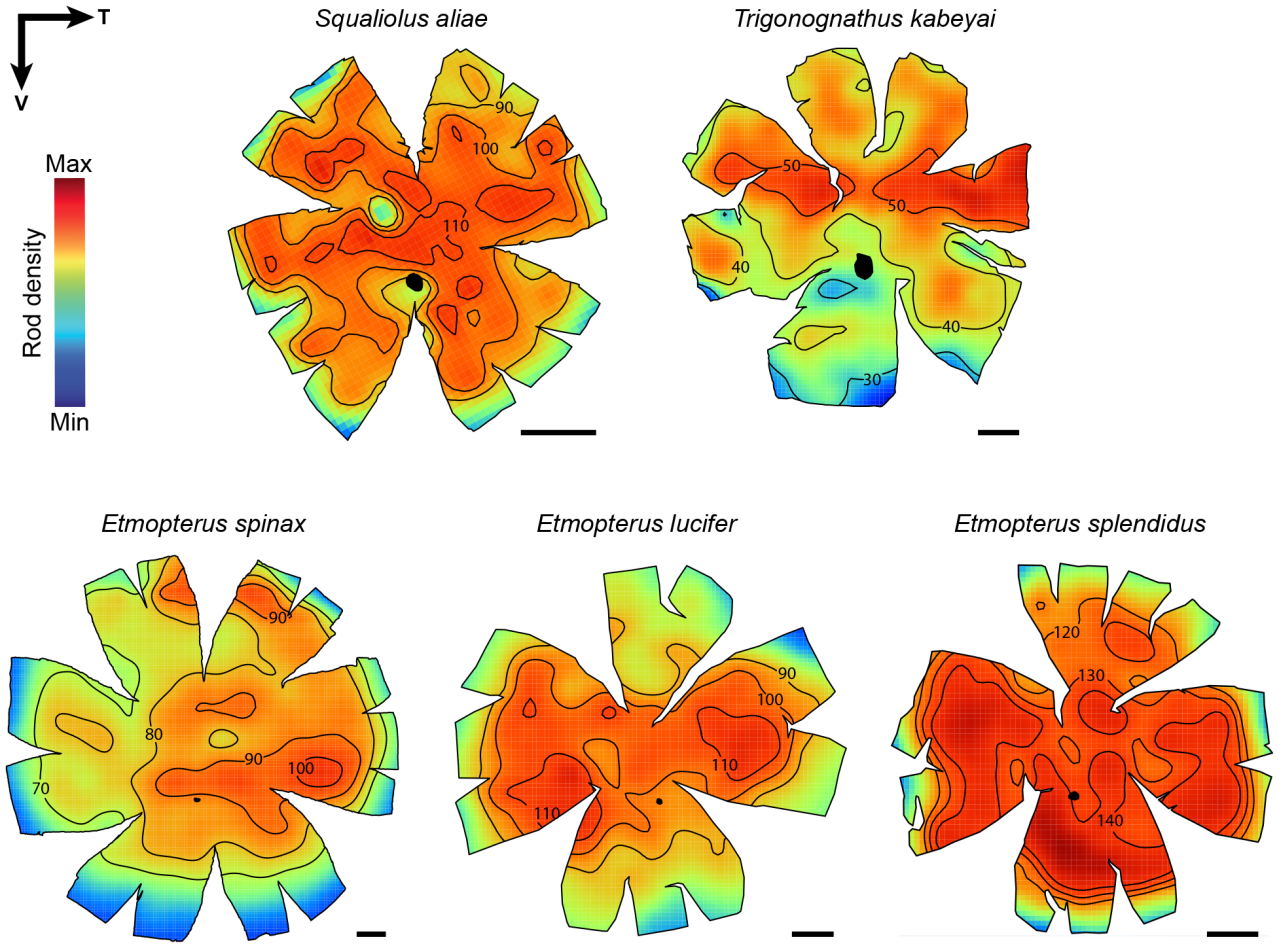
Topographic maps of photoreceptor densities. Black arrows indicate retina orientation (T = temporal, V = ventral). For comparative purpose, *T. kabeyai* retina (which comes from a left eye contrary to the other retinas) was vertically mirrored. Isodensity lines were arbitrarily selected in order to highlight the specialisations. All the densities are ×10^3^ cells mm^−2^. Scale bars, 2 mm. mm.

Although displaying shallow retinal density gradients, the rod photoreceptors demonstrate large interspecific variability in spatial distribution (Figure 3) with little variation within species. Pelagic species (*S. aliae* and *E. splendidus*) have a rather homogeneous retina with higher densities observed centrally although no clear specialisation can be distinguished. *Trigonognathus kabeyai* possesses a clear horizontal streak and an overall lower photoreceptor density than other species. *Etmopterus spinax* shows a well-defined temporal specialisation and a less defined dorsal area of higher photoreceptor density. *Etmopterus lucifer* possesses two areae arranged across the horizontal meridian, subtending both frontal and caudal regions of the visual field. Peak photoreceptor densities ranged from ∼67,000 rods mm^−2^ in *T. kabeyai* to ∼180,000 rods mm^−2^ in *E. splendidus* (Table 2).

**Table 2:** Photoreceptor cell (rod) data summary from retina wholemounts.

Species	TL (cm)	Eye Ø (mm)	Lens Ø (mm)	Total number (10^3^ cell retina^−1^)	Mean density (10^3^ cells mm^−2^)	Peak density (10^3^ cells mm^−2^)	Schaeffer CE* (site – rod numbers)†
*Etmopterus lucifer*	24.0^F^	8.29^R^	5.42	15211	100.5	147.2	0.0360 (196–9507)
*Etmopterus spinax*	40.0^F^	14.93^R^	7.14	37996	75.1	107.0	0.0175 (202–13358)
	48.0^F^	14.35^L^	8.40	29104	56.8	138.0	0.0370 (196–10232)
	37.0^M^	14.01^L^	7.31	30527	72.5	100.0	0.0250 (171–10732)
*Etmopterus splendidus*	20.3^M^	6.87^R^	3.87	15494	122.2	180.8	0.0220 (196–15131)
	22.2^F^	7.59^R^	3.77	16214	115.3	166.4	0.0210 (223–15834)
	24.2^F^	9.00^R^	4.46	16865	100.5	176.0	0.0290 (262–16470)
*Squaliolus aliae*	18.5^F^	5.90^R^	2.84	7488	103.1	152.0	0.0334 (207–13000)
	17.8^F^	6.10^R^	2.56	6876	95.6	149.0	0.0146 (200–11938)
*Trigonognathus kabeyai*	30.0^F^	7.08^L^	2.38	7801	41.0	67.2	0.0190 (189–4876)

*The Schaeffer CE measures the accuracy of the counting; it should be <0.1 to be acceptable [87], [88].

† Numbers for sites and rod indicate the total number of sampling sites and total number of counted rods, respectively. F, female; L, left eye; M, male; R, right eye; TL, total length.

**Figure 4:**
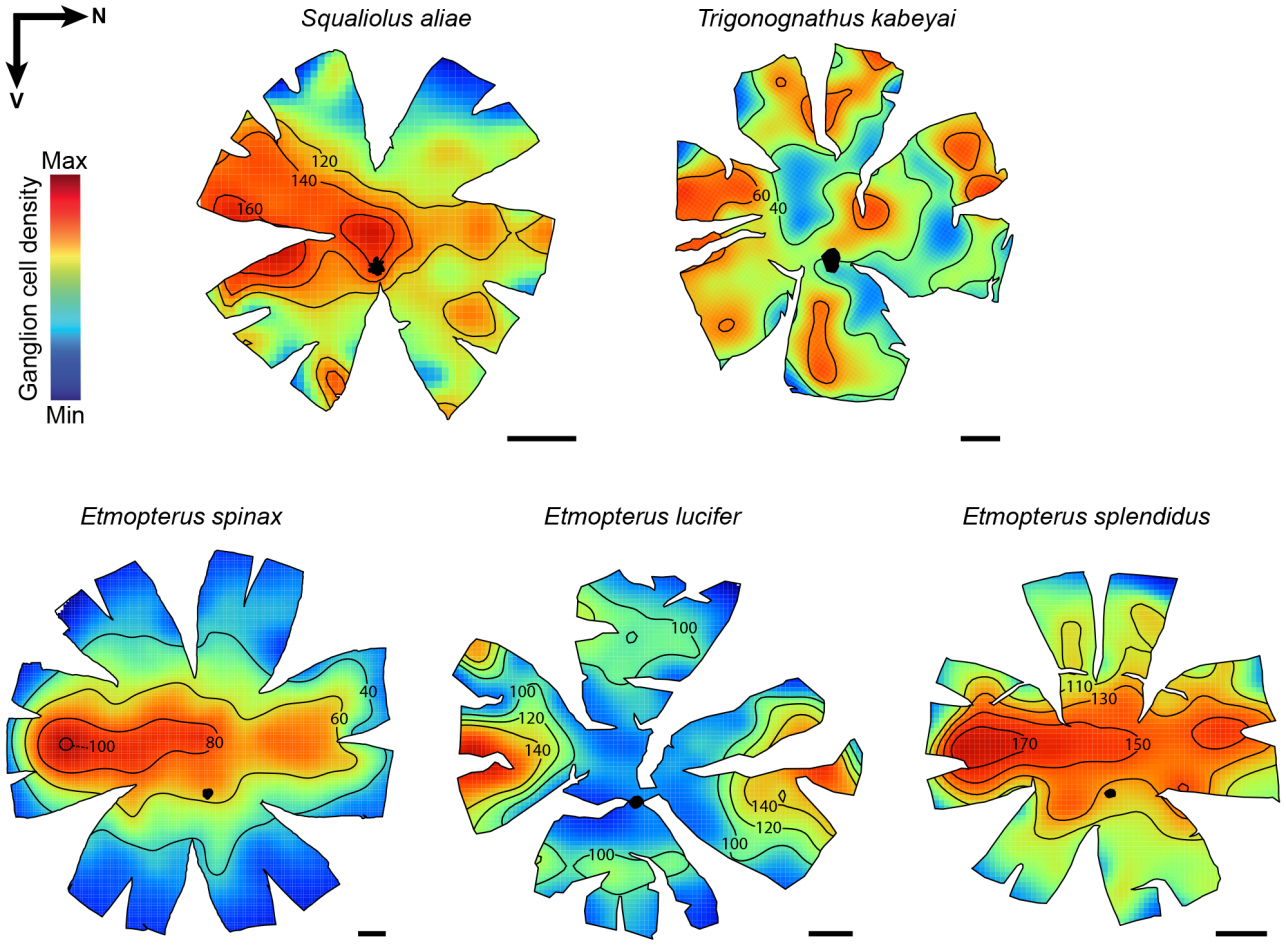
Topographic maps of ganglion cell densities. Black arrows indicate retina orientation (N = nasal, V = ventral). For comparative purpose, *T. kabeyai* retina (which comes from a left eye contrary to the other retinas) was vertically mirrored. Isodensity lines were arbitrarily selected in order to highlight the specialisations. All the densities are ×10 cells mm^−2^. Scale bars, 2 mm. mm.

The gradients of ganglion cell densities across the retina are shallow but, in contrast to the photoreceptor topography, there is less interspecific variation in cell density (Figure 4). *Squaliolus aliae*, *E. spinax* and *E. splendidus* have a temporal specialisation that is extended to include a nasal specialisation in *E. lucifer*. The ganglion cell distribution pattern is less clear in *T. kabeyai*, but seems to correspond to a dorsal arch-like specialisation subtending the lower frontal visual field. Several high-density patches can be found within the arch, the largest being located in temporal retina. Ganglion cells in these bioluminescent sharks show an overall low density, with peaks ranging from ∼900 cells mm^−2^ in *T. kabeyai* to ∼3900 cells m^−2^ in *S. aliae* (Table 3).

**Table 3:** Ganglion cell data summary from retina wholemounts.

Species	TL (cm)	Eye Ø (mm)	Lens Ø (mm)	Total number (10^3^ cell retina^−1^)	Mean density (cells mm^−2^)	Peak density (cells mm^−2^)	Schaeffer CE* (site – rod numbers)†
*Etmopterus lucifer*	24.0^F^	8.29^L^	5.42	153.6	1028.5	2533.4	0.0400 (169–5,761)
*Etmopterus spinax*	40.0^F^	14.93^R^	7.14	238.5	486.6	1066.7	0.0340 (197–3,494)
	48.0^F^	14.35^L^	8.40	228.5	468.8	1200.0	0.0370 (199–3,347)
	37.0^M^	14.01^L^	7.31	217.3	539.4	1173.3	0.0410 (171–3,183)
*Etmopterus splendidus*	20.3^M^	6.87^R^	3.87	131.6	1221.2	1893.4	0.0016 (190–7,713)
	19.8^M^	7.08^R^	3.69	128.8	1219.7	2053.4	0.0290 (183–7,545)
	18.3^F^	6.00^R^	3.50	128.5	1572.5	2666.7	0.0440 (155–7,531)
*Squaliolus aliae*	18.5^F^	5.90^R^	2.84	42.5	681.1	1410.1	0.0334 (200–4,391)
	17.8^F^	6.10^R^	2.56	75.7	1191.5	2080.0	0.0289 (197–7,882)
	21.8^F^	6.67^R^	3.14	132.4	2033.4	3893.4	0.0400 (213–13,788)
*Trigonognathus kabeyai*	30.0^F^	7.08^L^	2.38	87.3	482.1	888.9	0.0310 (198–1,965)

*The Schaeffer CE measures the accuracy of the counting; it should be <0.1 to be acceptable [87], [88].

† Numbers for sites and rod indicate the total number of sampling sites and total number of counted rods, respectively. F, female; L, left eye; M, male; R, right eye; TL, total length.

Interestingly, while most specialisations within the Etmopteridae are coincident with the choroidal tapetum lucidum, there is no such relationship for *S. aliae* in which the tapetum is restricted to the lower retina where rod photoreceptor density is low.

### Visual performances

The rod photoreceptors of species investigated in this study have an optical sensitivity ranged from ∼1.6 µm^2^ sr in *E. splendidus* to ∼4.1 µm^2^ sr in *E. spinax* (Table 4). Convergence ratio were calculated and ranged from ∼76 in *S. aliae* to ∼139 in *T. kabeyai*. This spatial summation theoretically increases optical sensitivity by about one order of magnitude. Conversely, these bioluminescent sharks are endowed with a spatial resolving power which ranges (in the peak density region) from ∼1.7 cycles deg^−1^ in *S. aliae* and *T. kabeyai* to ∼3.1 cycles deg^−1^ in *E. spinax* (Table 4).

**Table 4:** Visual performance parameters summary.

Species	*S* * (µm^2^ sr)	Σ_R_ (rods gc^−1^)	*SPR* ^†^ (cycles deg^−1^)	Nyquist frequency (cycles s^−1^)
*Etmopterus lucifer*	1.69	97.72	2.73	27.04
*Etmopterus spinax*	3.55	136.74	3.33±0.23	18.20
*Etmopterus splendidus*	1.40	84.22	2.22±0.05	25.53
*Squaliolus aliae*	2.09	76.31	1.81±0.37	26.65
*Trigonognathus kabeyai*	?	139.39	1.81	16.02

*These values are calculated for the specimen used in the histological analysis of photoreceptors (Table 1). ∑, summation ratio; N, sensitivity to bioluminescent point sources; S, optical sensitivity; SPR, spatial resolving power.

### Visual pigments

**Figure 5:**
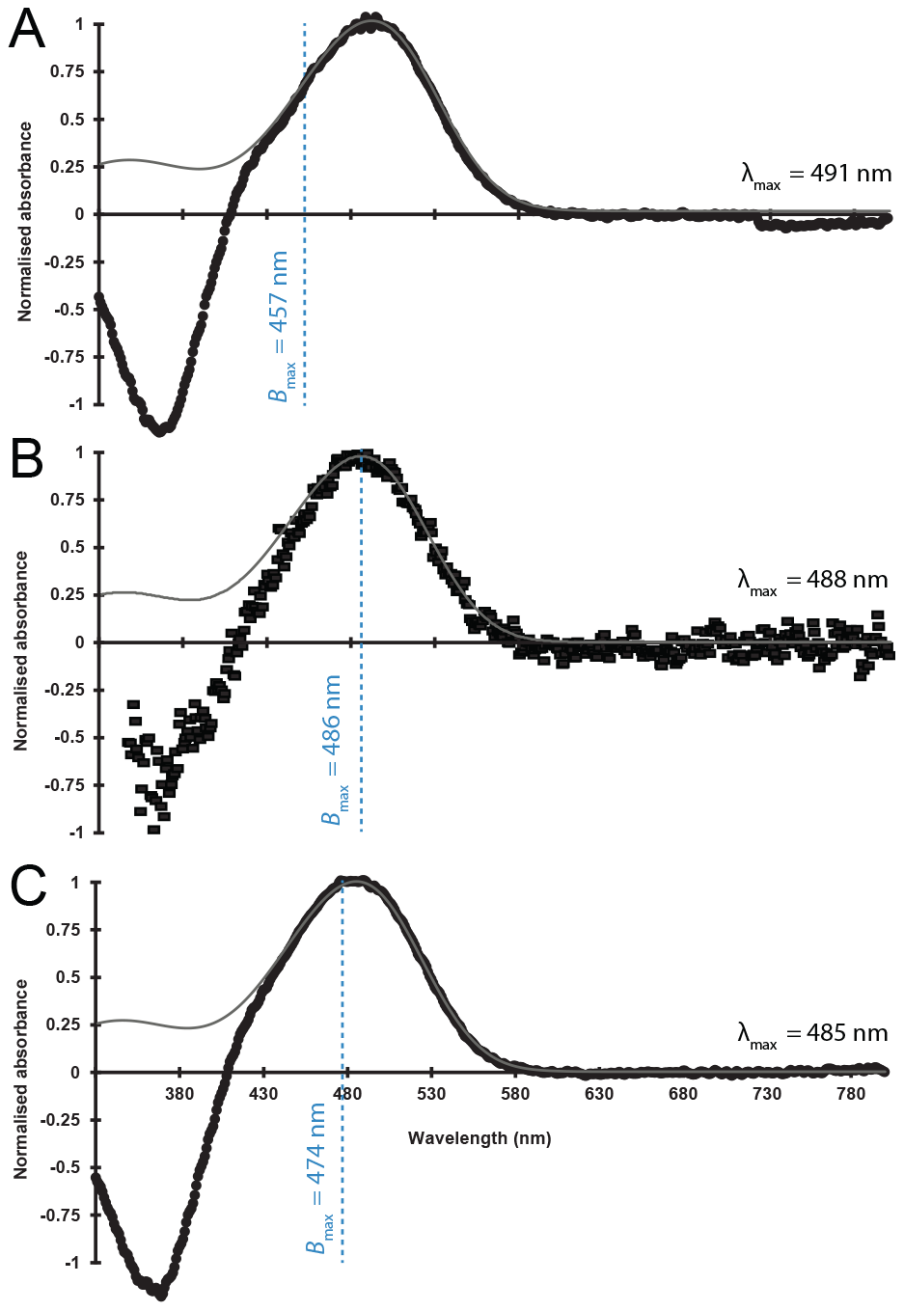
Rod photoreceptor spectral absorbance. Mean bleaching difference absorbance spectra (black symbols) with wavelength of maximum absorbance of the visual pigment (λ_max_; top for (A) *S. aliae*; (B) *E. spinax*; and (C) *E. splendidus*. Data for A and C were obtained by spectrophotometry of visual pigment extracts, that of B by microspectrophotometery (MSP). Absorption spectra are best fitted with visual pigment templates of appropriate λ_max_ (grey line) according to [86]. For comparison purpose, dashed blue lines at bioluminescence peak (*B*_max_) from [37] were superimposed on absorbance curves.

Only three species were available for visual pigment spectrophotometry (*E. spinax*, *E. splendidus* and *S. aliae*). The retina of each of these sharks appears to have only one spectrally distinct visual pigment (Figure 5) and this was confirmed by partial bleaching in *E. splendidus* and *S. aliae* (data not shown). Given the goodness-of-fit to visual templates [39] for all absorbance spectra, visual pigments likely contain only the vitamin A_1_ chromophore (rhodopsin). Wavelength of maximum absorbance (λ_max_) values ranged from 485 nm in *E. splendidus* and 487.5 nm in *E. spinax* to 491 nm in *S. aliae*.

### Comparison with other sharks

**Figure 6:**
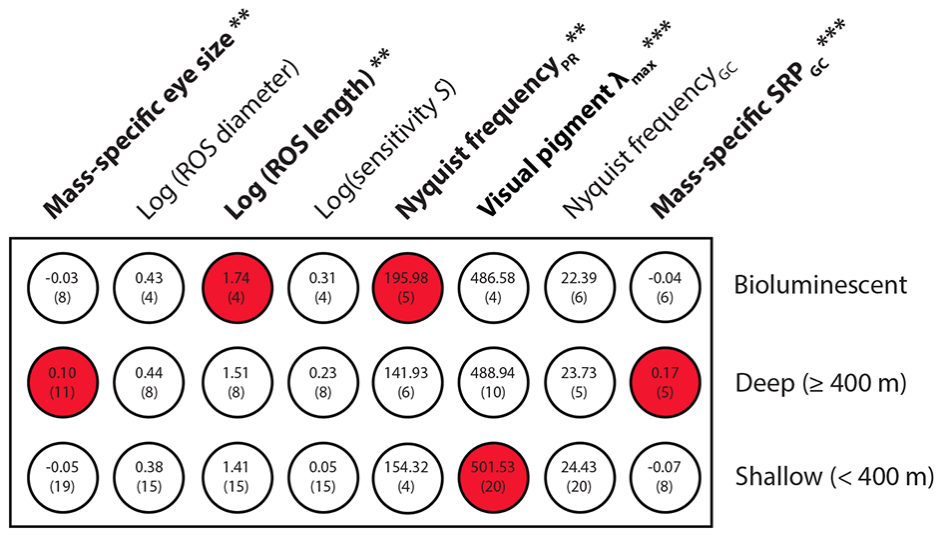
Comparative shark vision. Summary chart of statistical tests performed to compare the visual parameters of bioluminescent, deep living and shallow living sharks (see Dataset S1). When a significant difference between groups was detected by ANOVA (*P<0.05, **P<0.01, ***P<0.001), red colour was used to highlight the groups with statistically higher values (*P*<0.05 with post-hoc Student's *t*-test). Mean parameter values for each group are indicated into the corresponding circles. Values into brackets correspond to the number of species encompassed by each group.

Statistical analyses highlight numerous differences in mass-independent visual parameters between the three shark groups (‘bioluminescent’, ‘deep-living’ and ‘shallow-living’); only rod outer segment (ROS) diameter, photoreceptor optical sensitivity and ganglion cell Nyquist frequency (in the region of peak retinal photoreceptor density) remain uniform across the species (Figure 6). Overall, deep-sea species have a higher mass-specific eye size, which implies a relatively higher focal length and consequently a higher mass-specific spatial resolving power. Predictably, deep-sea species (which include bioluminescent species) have lower rod λ_max_ values than shallow water sharks: an adaptation to see the largely shortwave light present in their environment. Bioluminescent sharks have significantly longer ROS lengths and a higher photoreceptor Nyquist frequency (in the peak density region) than other shark groups.

## Discussion

This work aimed to investigate the visual system of five species of mesopelagic bioluminescent sharks. It reveals not only an unexpected diversity of photon capture strategies among this group but also a novel cranial structure –the etmopterid translucent tissue in the upper orbital region– and several other ocular specialisations once believed to be only found in bony fishes (Osteichthyes) such as aphakic gaps and semicircular tapeta. The discovery of these anatomical features, which are likely linked to the necessity to visualize bioluminescence in these fishes, emphasizes the current paucity of knowledge of deep-sea sharks, which represent a large part of shark biodiversity [27].

### Vision and bioluminescence

All sharks investigated in this study possess thousands of ventral photophores to counterilluminate i.e. to obliterate their silhouette from upward-looking animals deeper in the water column. To be efficient, however, this glowing camouflage has to be precisely controlled. In particular, the physical characteristics (i.e. spectral radiance distribution) of the emitted light needs to mimic that of the downwelling residual sunlight [29], [30]. Spectral tuning of luminescence is generally performed by biochemical (specialized light-emitting molecules sometimes combined with fluorescent compounds [40]) ]), and angular radiance tuning is generally achieved by physical means (i.e. optical filters and/or reflective structures [41]). Such tuning is facilitated by a mesopelagic light environment that has a radiance distribution that is virtually symmetrical about the vertical, and which has a predicable spectral range [42], [43]. Adaptations that allow occupants of this ‘twilight’ environment to match the intensity of the residual sunlight –which displays large temporal and depth-related variations in pelagic environments– are more complex because they involve a rapid feedback control mechanism of luminescence intensity. Many mesopelagic organisms including bony fishes, crustaceans (shrimps) and squids have large ocular photophores whose light emissions are directed towards the eye in order to allow comparison with the residual sunlight [44], [45]. More recently, the bacterial photogenic organs of a small squid (*Euprymna scolopes*) have been shown to contain extra-ocular photoreceptor molecules, which absorb and monitor the light produced by their symbionts, independent of the image-forming eyes [46]. In some species of bony fishes e.g. the lanternfish *Triphoturus mexicanus*, the ambient light intensity is monitored by photoreceptors protruding into the lumen of the pineal vesicle [47]. Such mechanisms have not yet been discovered in bioluminescent sharks, although the presence of a clear pineal window and the use of melatonin to control photophore emission by all species investigated in this group [31], [32], [33], [48] clearly suggest the involvement of the pineal vesicle in the luminescence control mechanism.

The translucent region of the upper orbit and its adjacent photophores discovered in all etmopterid species investigated in this study may represent a new kind of cranial structure analogous to the ocular photophores of other counterilluminating organisms, allowing comparison between the residual downwelling light (through the translucent tissue) and the photophore output. Alternatively, it could provide a preferential location to house spectral filtering tissue for breaking counterilluminating camouflage. Bioluminescent emissions tend to be spectrally broader than the surrounding daylight, with more light emitted towards the long wavelength (green) range of the emission peak. Many mesopelagic fishes use this subtle wavelength differential to detect counterilluminating animals using yellow lenses that act as long-pass filters [2], [4], [49], [50]. Sharks investigated in this study, however, have clear lenses that lack pigments absorbing in the human-visible spectrum, but further examination of the upper eye orbital tissue from fresh specimens is required to determine the filtering capabilities of the peculiar translucent area. The pygmy shark *S. aliae* lacks this orbital structure but shows a dorsal groove and a ventral aphakic gap that might function in an analogous way, facilitating comparison between downwelling light and bioluminescence produced by photophores adjacent to the eyes. Interestingly, all bioluminescent sharks investigated here possess a ventral retina with large photoreceptors and few ganglion cells (high spatial summation) and hence a high optical sensitivity to downwelling residual light, which suggests that this retinal area plays a major role in the counterillumination control mechanism.

Etmopterids display clade-specific lateral aggregations of photophores (Figure 1A) that are probably used in intraspecific communication [34], [35], [36]. It is therefore not unexpected that the visual systems of these sharks have co-evolved to optimize detection of these bioluminescent signals. In this context, the two acute zones of *E. lucifer*, which respectively subtend the nasal and temporal part of the visual field, likely play a role in the detection (photoreceptors) and localisation (ganglion cells) of conspecifics' bioluminescent flank markings. Importantly, the flank markings displayed by members of the *E. lucifer* clade are more nasally and caudally extended than those of other etmopterid clades [27], [36]. Such visual specialisations are remarkably similar to those of the bathypelagic tripod fish (*Bathypterois dubius*), which adopts a sit-and-wait strategy that requires concomitant monitoring of both the frontal and nasal parts of the visual field [13]. The smaller size of the lateral markings of the other investigated species of Etmopteridae suggests that the temporal area is solely responsible for the detection and localisation of these bioluminescent zones.

The visual pigments of the three species analysed in this study have absorption maxima (λ_max_) falling within the range of 484–491 nm. According to the ‘sensitivity hypothesis’ marine animals possess visual pigments with an absorption maxima (λ_max_) correlated with the peak wavelength of the residual downwelling light present in their environment [51], [52]. More recent studies suggest, however, that most visual pigments of deep-sea fishes are actually better adapted to see bioluminescence than downwelling sunlight (the optimal λ_max_ to see residual downwelling light is 474 nm) [9], [10], [53]. Bioluminescent sharks produce light with peaks within the blue region of the spectrum, although there is a large difference observed between the two families; dalatiid luminescence peaks lie at a considerably shorter wavelength (455 nm for *Isistius brasiliensis* [54] and 457 nm for *S. aliae* [55]) than etmopterid luminescence peaks (475 and 474 nm for the pelagic *E. splendidus* and *E. molleri*, respectively [55] and 486 nm for the coastal *E. spinax* [30]). Therefore, etmopterid luminescence matches the ambient downwelling light and falls into the classical range for bioluminescent organisms. As a consequence, their visual pigments appear relatively well adapted to see the light sources they can encounter in their habitat, including light produced by their conspecifics, which supports a putative bioluminescent communication mechanism. On the other hand, there is a large difference in the λ_max_ value (34 nm) between the visual pigment (491 nm) and the wavelength of peak luminescence of the pygmy shark, *S. aliae* (457 nm). This indicates that, although this species would certainly be able to perceive its own emission, its photoreceptors are spectrally tuned for the detection of other light sources such as other blue-emitting creatures on which it may prey [56] or even the downwelling sunlight. Indeed, *S. aliae* specimens analysed in this study were collected from a coastal turbid area where the ambient light is certainly displaced toward the long wavelength (green) range of the spectrum. The discrepancy between the λ_max_ of the visual pigment of the pygmy shark photoreceptor and luminescence emission supports the idea that dalatiid sharks, which lack any distinctive photophore markings, only use their luminescence for camouflage [55], [57].

### Vision and ecological niche

Beside their differences in luminescent capabilities, sharks investigated in this study also demonstrate a diversity of size (and hence vulnerability to predators), lifestyle and feeding strategies, which can severely impact the effectiveness of their visual system and how the eye perceive the environment [21], [22], [58], [59]. *Squaliolus aliae* and *E. splendidus*, which both possess small fusiform bodies, clearly have a pelagic habit [33], [56], [60], while *E. spinax*, *E. lucifer* and *T. kabeyai* are larger benthopelagic sharks [27], [60]. Observation and capture data indicate that at least some of these species undergo diel vertical migrations, probably to follow their prey [38], [56], [61], [62]. All species share a similar diet, which consists mainly of small mesopelagic fishes (mainly myctophids), crustaceans and cephalopods [27], [33], [63] although *E. spinax* is also known to feed on benthic and dead animals [64]. However, their teeth and jaw morphology display striking dissimilarities. *Trigonognathus kabeyai*, in particular, displays highly specialized triangular jaws endowed with needle-like teeth that contrast with the grasping/cutting dentition of other species. Although this has never been observed, these bizarre jaws (Figure 1) are likely to be rapidly projected forward to capture elusive prey, as is the case for the phylogenetically distant pelagic deep-sea goblin shark (*Mitsukurina owstoni*) with which it shares some similarities, including convergent evolution of its jaw structure [60], [65], [66].

### Specialisation for vision in specific regions of the visual field

The topographic differences observed across species are reflected in the function of the photoreceptors and ganglion cells. Photoreceptors initially encode light from an optical image of the visual environment, including any ecologically relevant visual stimulus (prey, predator or conspecific) passing across the visual field from all directions. Ganglion cells (the output cells conveying information to the visual centres of the brain), on the other hand, provide the ability to localise (spatially resolve) visual stimuli in a specific region of the visual field.

With respect to the topography of rod photoreceptors, this study has highlighted habitat-specific differences. Pelagic species display an almost homogeneous distribution of rods with no clear specialisations for “acute” (high sampling) vision, which is consistent with the need to detect visual stimuli from any direction in three-dimensional space. Benthopelagic species, on the other hand, show a continuum between an elongated temporal area (*E. spinax*) and a clear horizontal streak (*T. kabeyai*) although all gradients of rod density are quite shallow for each species.

The temporal rod photoreceptor specialisation of *E. spinax* may work in conjunction with the choroidal tapetum and the frontal aphakic gap in order to increase optical sensitivity in the frontal region of the visual field. Interestingly, the three specimens of *E. spinax* also showed a secondary dorsal arch-like specialisation of increased photoreceptor density. Such a retinal organisation facilitates the detection of moving objects in the inferior visual field [20], which certainly helps this shark to forage on the bottom, looking for benthic invertebrates (reptantid decapods, polychaetes and echinoderms) on which it is known to feed [20].

A horizontal streak allows a panoramic surveillance of a two-dimensional world, such as the sea bottom, with limited eye movements. This suggests that *T. kabeyai* displays a more benthic habitat than other investigated etmopterids. This benthic lifestyle is, however, probably only adopted during the daytime. Indeed, this species is often captured in the water column (sometimes near the surface) at night [38]; the only daytime capture events occur close to the bottom [61]. Moreover the extremely dense ventral photophore cover of the viper dogfish, the highest of any bioluminescent shark described thus far [55], indicates the necessity for this shark to use counterillumination, a camouflage technique typical of animals living in mesopelagic environments [29], [30], [45]. Overall, the bioluminescent sharks investigated here display quite different rod topographic patterns than those of the few other deep-sea shark species examined to date, which display temporal or central increases in rod density [17], [67].

In contrast to the situation for photoreceptor topography, the distribution of ganglion cells is similar across species i.e. an increase in ganglion cell density in the temporal area with various degrees of horizontal elongation and steeper density gradients; this pattern largely agrees with a previous, yet not stereology-based, description from the left eye of an *E. spinax* specimen [59]. Only *E. lucifer* reveals an additional nasal area almost certainly linked to the detection of the elongated photophore flank markings of its conspecifics, as previously discussed. Temporal specialisations are rare among sharks, which usually possess either a central area or a horizontal streak; it has only been reported in the bioluminescent cookiecutter shark *Isistius brasiliensis* [22], [59], [67], [68], [69], [70]. This general pattern may provide acute binocular vision and a higher visual sampling in the frontal part of the visual field [16], [17], [22]; thereby facilitating the detection of the bioluminescent emission pattern of conspecifics and/or the capture of small pelagic prey seen against in the darkness of the deep-sea. A similar function can be attributed to the series of acute areae that complement the temporal specialisation of *T. kabeyai* to form a dorso-temporal arch-like continuum that provides this shark with acute binocular vision in the ventro-frontal region of the visual field. This particular specialisation most likely allows this species to precisely evaluate the position of its prey and ensure a successful strike with its protrusible and raptorial jaws.

### Comparative study of shark visual system

Our comparative analysis of size-independent shark visual parameters is only exploratory since: (i) a small number of shark species were investigated (especially from the deep-sea), and (ii) the boundary (maximum recorded depth = 350 m) used to distinguish shallow and deepwater species is arbitrary and does not take into account the fact that some species encounter extremely variable light environments (during vertical movements or between photic and aphotic zones [2]). The dichotomy observed between deep and shallow living sharks for visual pigment λ_max_ value and relative eye size is nevertheless in accordance with the opposite requirements imposed by low and high light level habitats [67], [68], [69], which supports the validity of our approach.

Although no differences in ganglion cell density, which sets the upper limit of spatial resolving power and optical sensitivity [11], were detected across the different shark groups, bioluminescent sharks (which are all deep-sea species [27]) appear to possess longer rod outer segments (ROS) and to have higher rod densities (and thus smaller spatial rod Nyquist frequency) than other sharks. Members of Etmopteridae, with clade-specific lateral markings, also have a relative eye size similar to non-bioluminescent deepwater species. A long ROS and high eye-size: body-size ratio reflects a high sensitivity to bioluminescent point sources [2]. This indicates a strong necessity to detect all possible photons entering the visual field [1], a visual characteristic that has also been found in non-bioluminescent deep-sea sharks [65], [67].

The peak rod densities found in the visual specialisations of sharks investigated in this study are, on the other hand, exceptional among sharks and lead to summation ratios (76–139 photoreceptors per ganglion cell) clearly higher than estimates (25 to 50 photoreceptors per ganglion cell) from previous studies on other sharks (which include the deep-sea *Squalus mitsukurinii*) [22], [71]. Comparison with previous studies are, however, limited since the present study is the first to use stereology to assess both photoreceptor and ganglion cell densities in the same species/retina. Such high convergence ratios may be linked to the ability to detect bioluminescent signals. In addition to allowing high sampling of a visual scene, rod acute zones could also provide bioluminescent sharks with higher temporal resolution than other deep-sea sharks with pure rod retinas (which are typically correlated with ‘slow’ vision [11]). A higher temporal resolution could facilitate bioluminescent signalling within species of the Etmopteridae, which would require the capacity to detect and follow small glowing areas of conspecifics during dynamic behaviours such as cohesive swimming and hunting [34]. Future work, including visual modelling based on *in vivo* luminescence recordings as well as electrophysiological recordings of flicker-fusion frequency (FFF) in isolated retinas, will address this hypothesis in order to investigate further the evolutionary interaction between bioluminescence and the visual capabilities in deep-sea sharks.

## Materials and Methods

### Fish collection

Shark specimens analysed in this study were obtained from several sources. Specimens from four species were obtained as freshly moribund by-catch, either from Taiwanese fisheries operating off Donggang harbour [*S. aliae* and *T. kabeyai* (midwater nets at 50–400 m); *E. splendidus* (bottom trawls at 300–600 m); authorization for by-catch collection was given by the National Science Council (NSC 102-2621-B-291-002) and the National Museum for Marine Biology and Aquarium (BMMBA1031015)], or from an Australian governmental deep-sea campaign operating off Fremantle [*E. lucifer* (midwater trawl at 676–680 m); Campaign SS10/2005]. *Etmopterus spinax* specimens were collected in the Norwegian Raunefjord (bottom longlines at 180–200 m; Permit 12/14048) and humanely euthanized by a blunt trauma to the chondrocranium according to the local rules for experimental fish care (approval was given by the University of Bergen ethics committee). None of the authors are affiliated with the University of Bergen. However, *E. spinax* sacrifice and dissection was performed in a biological station close to capture site during the field trip. This station is affiliated to University of Bergen and hence we had to comply with their IACUC approvals. Specimens of *E. spinax* were killed by a quick blow to the head using a baton and head decapitation was subsequently performed to ensure death. All specimens were measured and sexed. Photographs (including close-ups) were taken from body and head in normal light.

### Retinal Topography

Eyes were isolated from their orbit, oriented by a dorsal cut and fixed in 4% formaldehyde in a specialized shark saline (292 mmol l^−1^ NaCl, 3.2 mmol l^−1^ KCl, 5 mmol l^−1^ CaCl2, 0.6 mmol l^−1^ MgSO4, 1.6 mmol l^−1^ Na2SO4, 300 mmol l^−1^ urea, 150 mmol l^−1^, trimethylamine N-oxide, 10 mmol l^−1^ glucose, 6 mmol l^−1^ NaHCO3 total osmolarity = 1080 mosmol, pH = 7.7 [72]) for a week and stored in 0.1 M phosphate buffer (PB, pH = 7.4).

Wholemounts were prepared according to standard techniques [68], [73] and were used either for photoreceptor topography, ganglion cell topography or both (in most cases; see technique from [74]). Cornea, lens, sclera, choroid and pigmented retinal epithelium (including the reflective tapetum lucidum) were carefully removed and peripheral slits were made in order to flatten the whole retina onto a glass slide. Several morphometric parameters were measured using callipers throughout the whole dissection process including pupil, lens and eye (axial direction) diameters. For measurement of photoreceptor topography, the photoreceptor layer was placed uppermost and the preparation was infiltrated by a few drops of glycerol (to increase optical contrast), mounted under a cover slip and sealed with nail varnish (to avoid dehydration). For ganglion cell topography, the retina was placed on a gelatinized slide with ganglion cell layer uppermost and dried in formalin vapour in two successive sessions (24 h/RT, 1 h/60°C) to increase cell differentiation before staining, which was performed according to Coimbra et al. [75]. The wholemount was then rehydrated, stained with acidified 0.1% cresyl violet for three minutes, dehydrated with an ethanol series, cleared in xylene and mounted in Entellan New (Merck, Germany).

Wholemounts were observed using a compound microscope (Optiphot-2, Nikon, Tokyo, Japan) equipped with a motorized stage (MAC200; Ludl Electronic Products, Hawthorne, USA) and a digital camera (Microfire; Optronics, Goleta, USA) and coupled to an IBM-PC compatible microcomputer running a stereological analysis software package (Stereo Investigator; MicroBrightField, Colchester, USA). The total number and topographic distribution of photoreceptors and ganglion cells were established using the optical fractionator method, treating wholemounts as single sections (thickness sampling factor = 1) [75], [76]. Cytological criteria from Hart et al. [77] were used to distinguish between ganglion cells and displaced amacrine cells: cells with large polygonal soma, abundant Nissl substance and a prominent nucleolus were considered to be ganglion cells while cells with a smaller and more circular profile, a lower cytoplasmic-to-nuclear volume ratio, less Nissl substance, and a more darkly stained nucleus were considered to be amacrine cells. After the contours of the retina and optic nerve were digitized, cells were counted using a convenient counting frame size (i.e. which allowed for significant changes in retinal density to be identified), and the systematic random grid spacing was adapted to reach a reduced coefficient of error (Schaeffer CE<0.05), which typically allowed for ∼200 sampling points per retina. High-resolution subsampling was performed in high-density ganglion cell areas to determine peak value and localisation. Cell count data were finally interpolated with R v. 2.15.2 to produce topographic maps (Thin Plate Spline model) following the protocol of Gisholt et al. [78].

### Photoreceptor morphology

The photoreceptor morphology of the different species was investigated by light microscopy in transverse semi-thin sections of the retina. Formaldehyde-fixed pieces of retina were post-fixed for one hour in 1% osmium tetroxide in 0.15 M PB, dehydrated in an ethanol and propylene oxide series and infiltrated with procure/araldite (ProSciTech Pty. Ltd., Townsville, Australia). Semi-thin sections (1 µm) were cut with a glass knife using an ultratome (LKB Ultratome Nova, LKB, Bromma, Sweden). Sections were stained with Toluidine blue, permanently mounted in Entellan (ProSciTech) and photographed using a digital camera mounted on a compound light microscope (Olympus BX50, Olympus Co. Ltd., Tokyo, Japan). For comparative purposes, morphometric measurements (outer/inner segment length, outer segment diameter) were then digitally measured using software Image J v. 1.46 in 10 rods from the central retina of all species.

### Spatial Resolving Power and Sensitivity Calculations

The spatial resolving power (*SRP*) characterizes the angular fineness with which an eye samples its visual environment [79]. For a hexagonal retinal mosaic, it is calculated (in cycles per degree) using the peak density of ganglion cells (*D*; in cells mm^−2^), following Hart et al. [77]:
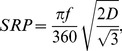
(2.1)where *f* is the focal length of the eye, which was considered in this study to be 2.75 times the lens radius, a typical value for elasmobranch eyes [58].

Optical sensitivity characterizes the relative capacity of the eye to capture light from a scene of uniform luminance [77]. It can be calculated (in mm^2^ sr or µm^2^ sr) following Land [80], assuming pupil (*A*) and lens diameter is equivalent:
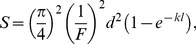
(2.2)where *F* is *F*-number (i.e. *f* ⋅ *A* ^−1^ and hence equals 1.375 for elasmobranch eye [58] while *d*, *l* and *k* are respectively the diameter, length and Napierian absorption coefficient of the photoreceptor outer segment. The absorption coefficient *k* was here fixed at 0.037 µm^−1^, which corresponds to a typical value for elasmobranch photoreceptors [81].

Mean summation ratio (Σ_R_), which indicates the mean number of photoreceptors subtended by each ganglion cell, was also calculated for each species by dividing mean photoreceptor density by the mean ganglion cell density, both obtained by averaging the available individual values.

### Visual Pigment Spectral Absorbance

Two different sampling techniques were used to measure visual pigment spectral absorbance.

#### Technique 1 (for E. splendidus and S. aliae)

Eyes were removed in darkness, placed in liquid nitrogen and stored at −80°C until dissection. Eyes were dissected and visual pigments were extracted in a 1 ml TRIS-buffered saline containing 100 µl of 200 mmol l^−1^ n-dodecyl b-D-maltoside, a mild detergent [82], under dim far-red illumination. Visual pigments were then partially bleached using the method of Douglas et al. [83]. Briefly, 5 µl of 1 mol l^−1^ hydroxylamine (NH_2_OH; pH 6.5) was added to 150 ml of dark-adapted extract and scanned in a Shimadzu UV2101-PC spectrophotometer. The sample was exposed to a series of bleaches using monochromatic light of decreasing wavelength from a regulated AC light source combined with narrow band interference filters (10 nm bandwidth B40 filters, Balzer, Liechtenstein) and rescanned between each bleach. One final exposure for 1 min in white light was used to ensure complete bleaching of any remaining visual pigment. Absorbance spectra were calculated as the difference spectra between sequential monochromatic partial bleaches, and a final difference spectrum was obtained by subtracting the final (bleached) scan from the initial scan.

#### Technique 2 (for E. spinax)

Eyes were dissected under dim red illumination to free the retina, which was subsequently fixed in 2% glutaraldehyde in the shark saline for 5 minutes and stored in 0.1 M phosphate buffer (pH = 7.4), a method inspired by [84]. Following the technique of Hart et al. [69], small pieces of retinal tissue were mounted in a drop of 310 mOsmol kg^−1^ PB saline (Oxoid, Basingstoke, UK) containing 8% dextran (D4876, Sigma Chemical Co., St. Louis, USA) and mounted between two coverslips. Transverse absorbance spectra (330–800 nm) of individual photoreceptor outer segments were measured using a single-beam wavelength-scanning microspectrophotometer [69], [77]. A measuring beam (∼1×3 µm) was aligned in an outer segment to provide a prebleach scan by recording the amount of light transmitted at each wavelength across the visible spectrum; a cell-free area of the preparation situated close to the outer segment was then used to provide a baseline scan. A broad-spectrum white light was used to bleach the outer segment for two minutes. Postbleach and baseline scans were performed to verify the presence of a photolabile visual pigment. For each outer segment, prebleach and postbleach spectra were subtracted to provide a bleaching difference absorbance spectrum.

All absorbance spectra were analysed following the methods of McNichol [85] and Govardovskii et al. [86] to provide an estimate of the wavelength of maximum absorbance (λ_max_) of the visual pigment. Visual absorbance spectra were then compared with the wavelength of peak bioluminescence emission (*B*_max_) available from the literature [37].

### Comparative shark vision

A dataset of size-independent visual parameters from 68 shark species was created using information from the literature (Dataset S1). Sharks were then classified into three categories using information from Ebert et al. [27]: (i) bioluminescent (sharks with light organs), (ii) deep (sharks always found below 350 m and (iii) shallow (sharks always found above 350 m). The visual parameters of these categories were finally compared using one-way analysis of variance (ANOVA). Normality and equality of variance were tested using Shapiro–Wilk and Levene's tests, respectively (data were log-transformed when these parametric assumptions could not be met). When a statistical difference was detected by ANOVA, we performed post-hoc Student's *t*-tests in order to test all pairwise comparisons. Statistical analyses were performed using the software JMP v.11 (SAS Institute Inc., Cary, NC, USA) and considered to be significant at the 0.05 level.

## Supporting Information

Dataset S1 **Supplementary dataset file containing shark photoreception data compiled from the literature and the present paper.** These data were used in comparative analyses of shark visual system whose results are presented in Figure 6.
(XLSX)

File S1 **References S90–S103.** File containing the supplementary references linked to some of the data present in the File S1.
(DOCX)
