# Supplementary material for: Photon Hunting in the Twilight Zone: Visual Features of Mesopelagic Bioluminescent Sharks
Source: PLoS One. 2014 Aug 6;9(8):e104213. doi: 10.1371/journal.pone.0104213 (PMC4123902; doi:10.1371/journal.pone.0104213)
Supplement: File S1 — References S90–S103. File containing the supplementary references linked to some of the data present in the File S1. (DOCX) [file pone.0104213.s002.docx]

**Supplementary references**

1. Bozzano, A (2004) Retinal specialisations in the dogfish *Centroscymnus coelolepis* from the Mediterranean deep-sea. *Scientia Marina* 68: 185-195.
2. Muguruma K, Shiro T, Yamamoto N (2013) Retinal ganglion cell distribution and spatial resolving power in the Japanese catshark *Scyliorhinus torazame*. *Zoological Science* 30: 42-52.
3. Gruber SH (1975) Duplex vision in the elasmobranchs: histological, electrophysiological and psychophysical evidence. In: Ali MA, editor. Vision in fishes: new approaches in research. London: Plenum Press. pp 525-540.
4. Harahush BK, Hart NS, Green K, Collin SP (2009) Retinal neurogenesis and ontogenetic changes in the visual system of the brown banded bamboo shark, *Chiloscyllium punctatum* (Hemiscyllidae, Elasmobranchii). Journal of Comparative Neurology 513: 83-97.
5. Gruber SH, Hamasaki DI, Bridges CDB (1963) Cones in the retina of the lemon shark (*Negaprion brevirostris*). Vision Research 3: 397-399.
6. Theiss SM, Collin SP, Hart NS (2010) Interspecific visual adaptations among wobbegong sharks (Orectolobidae). Brain, Behavior and Evolution 76: 248-260.
7. Denton EJ, Shaw TI (1963) The visual pigments of some deep-sea elasmobranchs. Journal of the Marine Biological Association of the United Kingdom 43: 65-70.
8. Crescitelli F, McFall-Ngai M, Horwitz J (1985) The visual pigment sensitivity hypothesis: further evidence from fishes of varying habitats. Journal of Comparative Physiology A 157: 323-333.
9. Sillman AJ, Letsinger GA, Patel S, Loew ER, Klimley AP (1996) Visual pigments and photoreceptors in two species of shark, *Triakis semifasciata* and *Mustelus henlei*. Journal of Experimental Zoology 276: 1-10.
10. Cohen JL, Hueter RE, Organisciak DT (1990) The presence of a porphyropsin-based visual pigment in the juvenile lemon shark (*Negaprion brevirostris*). Vision Research 30: 1949-1953.
11. Wald G (1939) The porphyropsin visual system. Journal of General Physiology 22: 775-794.
12. Beatty DD Visual pigments of three species of cartilaginous fishes. Nature 222: 285-285.
13. Hueter RE (1991) Adaptations for spatial vision in sharks. Journal of Experimental Zoology 5: 130-141.
14. Osmon AL (2004) The organization of the visual system in the bonnethead shark (*Sphyrna tiburo*). PhD Dissertation. University of South Florida.
